# Chicken TREM-B1, an Inhibitory Ig-Like Receptor Expressed on Chicken Thrombocytes

**DOI:** 10.1371/journal.pone.0151513

**Published:** 2016-03-11

**Authors:** Vanessa Turowski, Beatrice Sperling, Matthias A. Hanczaruk, Thomas W. Göbel, Birgit C. Viertlboeck

**Affiliations:** Institute for Animal Physiology, Department for Veterinary Sciences, University of Munich, Munich, Germany; Cornell University, UNITED STATES

## Abstract

Triggering receptors expressed on myeloid cells (TREM) form a multigene family of immunoregulatory Ig-like receptors and play important roles in the regulation of innate and adaptive immunity. In chickens, three members of the TREM family have been identified on chromosome 26. One of them is TREM-B1 which possesses two V-set Ig-domains, an uncharged transmembrane region and a long cytoplasmic tail with one ITSM and two ITIMs indicating an inhibitory function. We generated specific monoclonal antibodies by immunizing a Balb/c mouse with a TREM-B1-FLAG transfected BWZ.36 cell line and tested the hybridoma supernatants on TREM-B1-FLAG transfected 2D8 cells. We obtained two different antibodies specific for TREM-B1, mab 7E8 (mouse IgG1) and mab 1E9 (mouse IgG2a) which were used for cell surface staining. Single and double staining of different tissues, including whole blood preparations, revealed expression on thrombocytes. Next we investigated the biochemical properties of TREM-B1 by using the specific mab 1E9 for immunoprecipitation of either lysates of surface biotinylated peripheral blood cells or stably transfected 2D8 cells. Staining with streptavidin coupled horse radish peroxidase revealed a glycosylated monomeric protein of about 50 kDa. Furthermore we used the stably transfected 2D8 cell line for analyzing the cytoplasmic tyrosine based signaling motifs. After pervanadate treatment, we detected phosphorylation of the tyrosine residues and subsequent recruitment of the tyrosine specific protein phosphatase SHP-2, indicating an inhibitory potential for TREM-B1. We also showed the inhibitory effect of TREM-B1 in chicken thrombocytes using a CD107 degranulation assay. Crosslinking of TREM-B1 on activated primary thrombocytes resulted in decreased CD107 surface expression of about 50–70%.

## Introduction

A balanced immune reaction is important to avoid either insufficient or exaggerated immune responses. Therefore the cells interact via a network of either activating or inhibitory signals, which fine-tune the outcome of the immune response. Many of these cell-cell interactions are still poorly understood, but a group of so called immunoregulatory receptors are very likely to be involved in this process [1–3]. They are cell surface receptors with either activating or inhibitory signaling potential which are biochemically divided in two different groups and belong to either type II transmembrane C-type lectins [4] or type I transmembrane Ig-superfamily members [5]. Activating receptors have a short cytoplasmic tail without any signaling capabilities, but display a positively charged amino acid in the transmembrane region, which can be either arginine or lysine. This is associated with the negatively charged residue of an ITAM containing adaptor molecule, which is DAP12 or common γ chain. An ITAM is a so-called immunoreceptor-tyrosine based activating motif, which is phosphorylated after receptor crosslinking and triggers intracytoplasmic activation cascades [6, 7]. The inhibitory receptors have an uncharged transmembrane region, but a long cytoplasmic tail with a different number of immunoreceptor-tyrosine-based-inhibitory motifs (ITIMs). Interaction with the specific ligand leads to phosphorylation of these tyrosines and subsequent recruitment of tyrosine specific protein phosphatases like src-homology phosphatase-1 (SHP-1), SHP-2 or Src homology 2-containing inositol 5'-phosphatase (SHIP) which are dephosphorylating downstream target molecules [8, 9].

Immunoregulatory Ig-like receptors are also present in chickens. One family are the chicken Ig-like receptors (CHIR), which were discovered in 2000 [10] and characterized in recent years by us and other groups [11–18].

The availability of the first assembly of the chicken genome in 2004 [19] offered the opportunity to further investigate additional immunoregulatory Ig-like receptor families by extended homology searches. By this method, we characterized the TREM (triggering receptors expressed on myeloid cells), SIRP (signal-regulatory proteins), CD200R (CD200 receptor family) and CD300L (CD300 antigen like family members) [20–23].

The chicken TREM family is located on chicken chromosome 26. It comprehends of one potentially activating receptor TREM-A1 and two potentially inhibitory receptors TREM-B1 and TREM-B2 [20]. TREM-A1 consists of a single V-set Ig-domain, a charged transmembrane region and a short cytoplasmic tail. TREM-B1 and TREM-B2 both display two extracytoplasmic V-set Ig-domains, an uncharged transmembrane region and a long cytoplasmic tail. The cytoplasmic tail of TREM-B1 encodes for one ITSM (immunoreceptor-tyrosine switch motif) and two ITIMs, whereas TREM-B2 only has two ITIMs. Interestingly, for TREM-B2 we cloned two splice variants either with one or two extracellular Ig-domains followed by the transmembrane and cytoplasmic region [20]. At that time the chromosomal region encoding the TREM family members was still unassembled in the chicken genome. Meanwhile we analyzed a completely sequenced BAC clone which included all TREM members and the unrelated flanking genes, which we used, apart from other characteristics, for assigning synteny to corresponding mammalian receptors. This is *gallus gallus* BAC clone CH261-94J19 (Acc. No.: AC161468). In addition to our initial characterization, we found that the TREM-B2 gene displayed four extracytoplasmic V-set Ig-domains, which probably arose by duplication of a two domain receptor, because they were identical to each other by 97.4% on nucleotide level. We verified this third splice variant for TREM-B2 by cDNA cloning (Viertlboeck et al., unpublished).

Recently, we examined the expression pattern of the activating TREM-A1 by generating a specific monoclonal antibody (mab) [22]. We showed high TREM-A1 expression on blood and bone marrow monocytes, macrophages, heterophils (which are the avian homologues to neutrophils) and on a newly discovered population of blood NK cells [24]. Furthermore we detected lower TREM-A1 expression on thrombocytes and B and T cell subsets. Additionally, we found TREM-A1 expression also in chicken brain cells, including microglial cells, neurons, astrocytes and oligodendrocytes.

In this report, we show detailed expression analysis of TREM-B1 employing two newly established mab. We found TREM-B1 to be expressed on chicken thrombocytes. Furthermore we used the specific mab to immunoprecipitate TREM-B1 protein from cell lysates of either PBMC or stably transfected 2D8 cells and we used this cell line to show that the tyrosine residues in the cytoplasmic region can be phosphorylated and recruit SHP-2. Moreover, we demonstrated that crosslinking of TREM-B1 resulted in a reduced surface expression of the degranulation marker CD107 on activated thrombocytes.

## Materials and Methods

### Ethics statement

All of the experimental procedures were in accordance with institutional, state and federal guidelines on animal welfare. The animal experiments were approved by the committee for the Care and Use of Laboratory Animals of the Government of Upper Bavaria, Germany (permit number: 55.2-1-54-2531.6–12.09) and all efforts were made to minimize animal suffering during experiments.

### Animals

Chicken line M11 was kindly provided by S. Weigend (Federal Research Institute for Animal Health, Mariensee, Germany). Fertilized Eggs were incubated and hatched at the Institute for Animal Physiology, University of Munich. The animals were housed under conventional conditions and experiments were performed at the age of 3 to 10 weeks. Balb/c mice were obtained from Charles River Wiga GmbH (Sulzfeld, Germany) and raised at the institute.

### Cell preparation

Leukocytes of chicken bursa, caecal tonsils, spleen and thymus were obtained by passing the organs through a stainless steel mesh and subsequent density centrifugation of the single cell suspension on Biocoll Separating Solution (Biochrom AG, Berlin, Germany). Heparinized whole blood was used to isolate either peripheral blood lymphocytes (PBL) by slow speed centrifugation [24] or peripheral blood mononuclear cells (PBMC) by density centrifugation [25]. Preparation of monocyte-derived macrophages was performed by in vitro culture of PBMC in RPMI 1640 (Biochrom AG, Berlin, Germany) supplemented with 8% FCS (Biochrom AG, Berlin, Germany) and 2% chicken serum (Life Technologies, Carlsbad, CA, USA) for 2 days (40°C, 5% CO_2_) [26]. For whole blood analysis by flow cytometry, EDTA-treated blood was diluted in PBS supplemented with 1% BSA and 0.01% NaN_3_.

### Cloning procedures

The expression constructs established in this study were generated by using cDNA templates obtained in a previous study [20] and specific oligonucleotides are summarized in Table 1. For the TREM-B1-FLAG construct, primers 1791 and 1792 were used on a TREM-B1 cDNA (Acc. No.: AM076722). The resulting PCR fragments were gel extracted and coupled to a modified pcDNA3.1/V5-His TOPO Vector (Life Technologies, Carlsbad, CA, USA) containing an N-terminal FLAG epitope tag by using the Gibson Assembly^™^ Master Mix (New England BioLabs Inc., Massachusetts, USA). The TREM-B1-FLAG-muCD3ζ expression construct was generated as the TREM-A1-FLAG-muCD3ζ construct described previously [22]. Briefly, the respective extracellular domains were amplified by PCR with primers 1148 and 1149, gel purified and EcoRI digested followed by ligation into a modified pcDNA3.1/V5-His TOPO Vector (Life Technologies, Carlsbad, CA, USA) resulting in an N-terminally FLAG-tagged extracellular region of TREM-B1 fused to the transmembrane region of chicken CD8α and the cytoplasmic domain of murine CD3ζ. For generation of the TREM-B2-FLAG-muCD3ζ construct, the same cloning vector was used, but ligation of the two extracellular Ig-domains, which were amplified by primers 1748 and 1748 on TREM-B2 cDNA (Acc. No.: AM076723), was performed with the Gibson Assembly^™^ Master Mix. Accuracy of the constructs was verified by gene sequencing (GATC, Konstanz, Germany).

**Table 1:** Oligonucleotides used for cloning.

Specificity	Number	Sequence^b^
TREM-B1-FLAG construct	1791s^a^	GGACGATGACGATAAGGCAGGAGAGGACACGCAAG
TREM-B1-FLAG construct	1792as	AGAATTGCCCTTGAACTTCTATAGGGTTGTGTCCT
TREM-B1-FLAG –muCD3ζ	1148s	ATGAATTCGCAGGAGAGGACACG
TREM-B1-FLAG –muCD3ζ	1149as	ATGAATTCAGCATAGGGGGTCCT
TREM-B2-FLAG –muCD3ζ	1748s	GGACGATGACGATAAGGGTCTCCCAGCCCAAACAG
TREM-B2-FLAG –muCD3ζ	1749as	TGGATATCTGCAGAATTTTGTGATGGTGTTCCGTG

^a^Orientation indicated as s sense and as antisense

^b^ Restiction site underlined

### Cell lines, transfection and expression

The mouse thymoma cell line BWZ.36 [27] was stably transfected by electroporation [28] using 25 μg of TREM-B1-FLAG-muCD3ζ (3 x 10^6^ cells, 200 V with 950 μF capacitance). Transfected cells were seeded into a 96-well flat bottom plate and selected in RPMI 1640 (Biochrom AG, Berlin, Germany) supplemented with 10% FCS and 0.8 mg/ml G418 (Biochrom AG, Berlin, Germany) for 10 days (37°C, 5% CO_2_). The chicken B cell line 2D8 [29] was stably transfected with a full-length TREM-B1-FLAG construct using Metafectene Pro (Biontex, Planegg, Germany). After incubation of 24 h (40°C, 5% CO_2_) the transfected cells were plated in a 96-well flat bottom plate and cultured in RPMI medium containing 10% FCS and 0,8 mg/ml G418 (Biochrom AG, Berlin, Germany) for 2 weeks. Single clones were screened for surface expression by anti-FLAG staining and subsequent flow cytometry (FACSCanto II, BD, Heidelberg, Germany). A BWZ.36 cell line stably expressing TREM-A1-FLAG-muCD3ζ was established in a previous study [22]. In addition, human embryonic kidney HEK 293 T cells were transiently transfected with a TREM-B2-FLAG-muCD3ζ construct using the Metafectene liposomal transfection reagent (Biotex, Planegg, Germany) and used for further analysis after 24 h.

### Real-time RT-PCR

RNA preparation and real-time RT-PCR was performed as described previously [22]. Total RNA of bursa, thymus, caecal tonsils, liver, spleen, bone marrow and PBMC was extracted from 100 mg tissue or 1 x 10^7^ cells by using Trizol (Life Technologies, Carlsbad, CA, USA). The RNA quality was determined with the 2100 Bioanalyzer (Agilent Technologies, Waldbronn, Germany) and RNA with an integrity number abover 7.5 was used for cDNA synthesis with the QuantiTect Reverse Transkription Kit (Qiagen, Hilden, Germany). PCR was performed with the PowerSYBR^®^ Green RT-PCR Reagents Kit (Applied Biosystems, Darmstadt, Germany) using the 7300 Real-Time PCR System (Applied Biosystems, Darmstadt, Germany) with following parameters: 95°C for 10 min, then 40 cycles of 95°C for 15 s, and 59°C for 1 min with subsequent analysis by a melting curve. The cDNA samples were analyzed in triplicates with oligonucleotides specific for TREM-B1 and 18S rRNA (Table 2) obtaining the cycle thresholds (Ct) for each tissue. The relative amounts of gene-of-interest mRNA were calculated by means of the ΔΔCt method as described before [21].

**Table 2:** Oligonucleotides used for real-time RT-PCR.

Specificity	Number	Sequence
TREM-B1	1060s^a^	ATTGGTCCTAACCGTGCACTGTA
TREM-B1	1061as	ATGTGCCAGAATCCTCTTTTCG
18S rRNA	870s	CATGTCTAAGTACACACGGGCGGTA
18S rRNA	871as	GGCGCTCGTCGGCATGTATTA

^a^Orientation indicated as s sense and as antisense

### Generation of a specific monoclonal antibody

Two monoclonal antibodies with different isotypes were generated as described previously [11]. Briefly, a Balb/c mouse was repeatedly immunized with the TREM-B1-FLAG-muCD3ζ transfected BWZ.36 cells. Hybridoma supernatants were screened by flow cytometry on TREM-B1-FLAG transfected 2D8 cells thereby excluding nonspecific mab binding to irrelevant BWZ.36 proteins. For additional verification of the mab specificities, the supernatants were also tested on the TREM-B1-FLAG-muCD3ζ transfected BWZ.36 cells and on both untransfected BWZ.36 and 2D8 cells. Cross-reaction with other structurally similar TREM members was examined by staining TREM-A1-FLAG-muCD3ζ transfected BWZ.36 and TREM-B2-FLAG-muCD3ζ transfected 293T cells. For our further analysis of TREM-B1, we selected the mab 1E9 (mouse IgG2a) and 7E8 (mouse IgG1). For whole blood staining, the mab 1E9 was affinity purified by protein G coupled agarose, subsequently concentrated using centrifugal filters (Merck Millipore, Darmstadt, Germany) and conjugated to the Alexa Fluor 647 dye (Life Technologies GmbH-Carlsbad, CA, USA) according to the manufacturer´s protocol.

### Antibodies and immunofluorescence analysis

Single-cell staining was performed with either the 1E9 or the 7E8 mab followed by a goat-anti-mouse IgG2a- or IgG1-PE conjugate (SBA, Birmingham, AL, USA). For double staining of leukocytes, cells were first incubated with a mixture of primary mab. Depending on the isotype of the other mab in the mixture either the anti-TREM-B1 mab 1E9 (IgG2a) or 7E8 (IgG1) was used. Subsequently, cells were incubated with a combination of goat-anti-mouse IgG2a-PE and goat-anti-mouse IgG1-FITC (SBA, Birmingham, AL, USA) when the 1E9 mab was used or a mixture of goat-anti-mouse IgG2a-FITC and goat-anti-mouse IgG-1-PE when 7E8 was used for staining. Following mab were used for further characterization of TREM-B1 expressing cells: 8G8 (mouse IgG2a) specific for CLEC-2, which is expressed on thrombocytes [30], K1 (mouse IgG2a) recognizing an unidentified antigen expressed on thrombocytes and macrophages [31], AV20 (mouse IgG1) specific for Bu-1 present on B cells [32], CT3 (mouse IgG1) specific for CD3 on T cells [33], KUL01 (mouse IgG1) specific for monocytes and macrophages [34], 8F2 putatively recognizing a CD11c homologue expressed on chicken thrombocytes, T cells, NK cells, monocytes, heterophils and eosinophils [24] and 8D12 specific for a chicken FcY receptor (CHIR-AB1) expressed on B cells, monocytes and macrophages, NK cells and heterophils [13, 22]. For each staining, appropriate isotype-matched controls were used. Dead cells were identified by staining with 7-AAD (7-aminoactinomycin D, Sigma-Aldrich, St. Louis, MO, USA) and the vital leukocyte population was analyzed by flow cytometry (FACSCanto II, BD, Heidelberg, Germany) using the BD FACS DIVA 6.1.3 and FlowJo Software (Tree Star inc., Ashland, OR, USA). Whole blood staining was performed with the Alexa Fluor 647-conjugated 1E9 mab combined with a phycoerythrin-conjugated K1 mab [31] and analyzed by flow cytometry as described previously [35].

### Biotinylation and pervanadate treatment of cells

Biotinylation and pervanadate treatment of cells were performed as described previously [11]. For biotinylation, 5 x 10^7^ of TREM-B1 transfected 2D8 cells or 1 x 10^8^ prepared PBMC were washed in PBS containing 1 mM MgCl_2_ and 0.1 mM CaCl_2_. After the cells were incubated in PBS with 0.5 mg/ml sulfo-NHS-biotin (Life Technologies, Carlsbad, CA, USA) for 40 min at 4°C under constant rotation, the cells were washed once in RPMI 1640 followed by two washes in PBS with MgCl_2_ and CaCl_2_. Efficiency of biotinylation was checked by flow cytometry using phycoerythrin-conjugated streptavidin (SBA, Birmingham, AL, USA). Pervandate treatment was conducted with 5 x 10^7^ cells in 5 ml of warm (37°C) medium. After stimulation for 0, 5 and 15 min with 0.1 mM Na_3_VO_4_ with 0.05% H_2_O_2_ at 37°C, cells were immediately pelleted by centrifugation at 4°C and washed twice in cold (4°C) PBS containing 0.4 mM EDTA and 0.4 mM Na_3_VO. Subsequently, cells were lysed for 45 min on ice using a lysis buffer consisting of 150 mM NaCl, 40 mM TRIS-Cl pH 7.4 and 1 mM EDTA supplemented with 1% TritonX-100, the protease inhibitor cOmplete (Roche, Basel, Switzerland) and in case of pervandate stimulation phosphatase inhibitors (10 mM NaF, 2 mM EDTA, 1 mM Na_3_VO_4_). Supernatant was separated from nuclear and insoluble components by centrifugation for 30 min at 4°C and 16,000 x g and subsequently used for immunoprecipitation.

### Immunoprecipitation, deglycosylation and immunoblotting

Immunoprecipitation was performed with 50 μl of either anti-Flag M2 Agarose Affinity Gel (Sigma-Aldrich, St. Louis, MO, USA) or protein G-coupled agarose (Merck Millipore, Darmstadt, Germany) loaded for 4 h with 1.5 μg of the purified 1E9 mab and washed once with lysis buffer before use. Lysates were added to the agarose-beads and incubated over night at 4°C under constant rotation. The beads were washed three times in lysis buffer by centrifugation before immunoprecipitates were eluted by boiling the beads in 100 μl of SDS sample buffer. For deglycosylation, immunoprecipitates were dissolved in a glycoprotein denaturing buffer followed by incubation in a buffer containing peptide N glycosidase F (PNGase, New England BioLabs, Ipswich, MA, USA) according to the manufacturer´s protocol. Precipitated proteins were separated on 12% SDS-PAGE under nonreducing and reducing conditions followed by transmission on a 0.45 μm nitrocellulose membrane (Amersham Hybond-ECL, GE Healthcare, Solingen, Germany) by semidry electroblotting. The blots were blocked in 5% nonfat dry milk in PBS supplemented with 0.05% Tween and subsequently incubated with a streptavidin-HRP (horseradish peroxidase) conjugate (1:10000, SBA, Birmingham, AL, USA), an anti-FLAG-POD (peroxidase) conjugate (1:20000, Sigma-Aldrich, St. Louis, MO, USA) for loading control or a HRP conjugated anti-phosphotyrosine antibody (P-Tyr (Py20) HRP, 1:200, Santa Cruz Biotechnology, Dallas, TX, USA) for phosphotyrosine detection. Phosphatase detection was conducted by using a rabbit anti-human-SHP-2 antiserum (SH-PTP 2 (C-18), 1:200, Santa Cruz Biotechnology, Dallas, TX, USA) followed by goat anti-rabbit IgG(H+L)-HRP (1:10000, Jackson ImmunoResearch Inc., West Grove, PA, USA). Immunoblotted proteins were visualized using a luminol-based chemiluminescent substrate. This consists of three components: ECL-A solution containing 0.1 M TRIS-HCl ad 100 ml aqua dest. pH 8.6 supplemented with 25 mg Luminol (Sigma-Aldrich, St. Louis, MO, USA), ECL-B solution comprised of 0.110 g parahydroxy coumarin acid (Sigma-Aldrich, St. Louis, MO, USA) solved in 100 ml DMSO and ECL-C solution which is H_2_O_2_ 30% (v/v).

### CD107 degranulation assay

To investigate the inhibitory potential of TREM-B1, the CD107 degranulation assay was performed [36]. 5 x 10^5^ PBMC were incubated simultaneously with the CLEC-2 specific mab 8G8 (mouse IgG2a) and the TREM-B1 specific mab 1E9 (mouse IgG2a), a goat-anti-mouse IgG2a-FITC conjugate (SBA, Birmingham, AL, USA) for co-crosslinking and the 5G10 mab detecting chicken CD107 (mouse IgG1, Developmental Studies Hybridoma Bank, University of Iowa) in the presence of the protein transport inhibitor GolgiStop^™^ (BD, Heidelberg, Germany). An appropriate isotype-matched control was performed identically, but in the presence of an irrelevant mouse IgG2a antibody instead of the 1E9 mab. Following incubation for 30 min (40°C, 5% CO_2_), the cells were stained with an anti-IgG1-PE antibody (SBA, Birmingham, AL, USA). Dead cells were discriminated using 7-AAD (7-aminoactinomycin D, Sigma-Aldrich, St. Louis, MO, USA) and living cells were analyzed by flow cytometry. Additional controls without crosslinking were incubated in the absence of 8G8 and 1E9 or the irrelevant antibody and stained with the respective antibodies at the end of the experiment. To determine the percentage of TREM-B1 induced inhibition, the fraction of CD107 positive cells out of the whole thrombocyte population was calculated and referred to the isotype-matched control, which were set to 100% degranulation. Statistical analysis was perfomed by using Graph Pad Prism software version 5.

## Results

### The mab 1E9 and 7E8 are specific for TREM-B1 and do not cross-react with other TREM members

**Fig 1:**
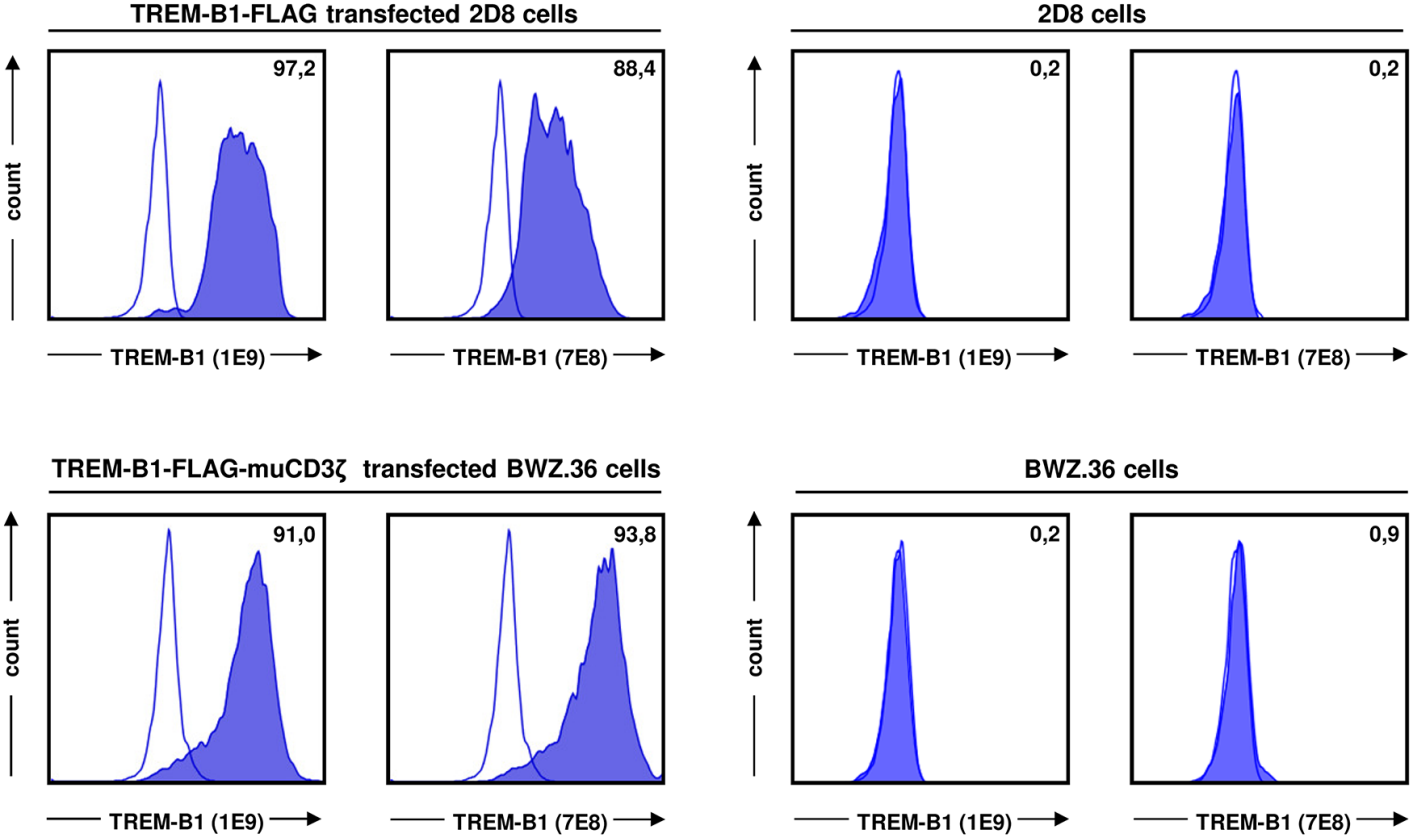
1E9 and 7E8 are specifically recognizing TREM-B1 protein. TREM-B1-FLAG transfected 2D8 and untransfected 2D8 cells were incubated with either the 1E9 or the 7E8 mab (filled histograms) and analyzed by flow cytometry (upper panels). TREM-B1-FLAG-muCD3ζ transfected BWZ.36 and untransfected BWZ.36 cells were tested in the same way (lower panels). Isotype-matched controls are shown as open histograms and percentages of positive cells are indicated as numbers.

In order to generate a monoclonal antibody against the extracellular domain of TREM-B1, two different cell lines were established. Immunization was conducted with a TREM-B1-FLAG-muCD3ζ transfected BWZ.36 cell line followed by screening of the hybridoma supernatants on chicken 2D8 cells stably expressing TREM-B1-FLAG (Fig 1). By this strategy, nonspecific mab detecting irrelevant BWZ.36 proteins were excluded. Two specific mab with different isotypes were selected, designated 1E9 (mouse IgG2a) and 7E8 (mouse IgG1). For validation of specificity, the obtained mab 1E9 and 7E8 were also tested on untransfected 2D8 and BWZ.36 cells as well as on TREM-B1-FLAG-muCD3ζ transfected BWZ.36 cell line (Fig 1). Both antibodies bound to nearly all transfected cells, whereas unspecific binding to the untransfected control cells could be excluded.

**Fig 2:**
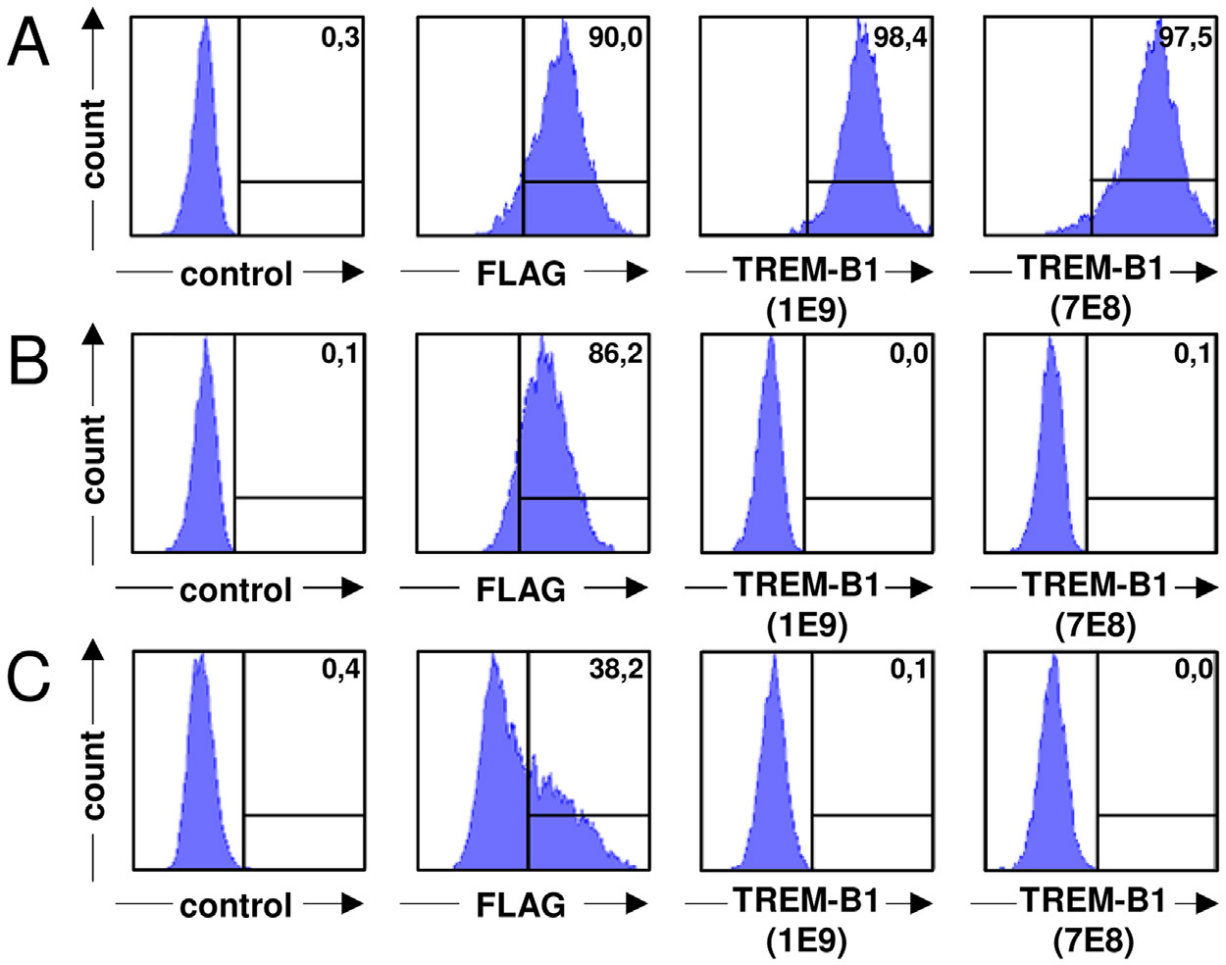
The mab 1E9 and 7E8 do not cross-react with other TREM family members. BWZ.36 cells stably expressing TREM-B1-FLAG-muCD3ζ (A) or TREM-A1-FLAG-muCD3ζ (B) and transiently transfected HEK-293T cells expressing TREM-B2-FLAG-muCD3ζ (C) were stained with an isotype-matched negative control (left panels), an anti-FLAG mab as expression control (middle panels, left) and the mab 1E9 (middle panels, right) and 7E8 (right panels). Numbers indicate the percentage of positive cells.

To test whether the mab could cross-react with other members of the chicken TREM family, 1E9 and 7E8 supernatants were also tested on TREM-A1-FLAG-muCD3ζ and TREM-B2-FLAG-muCD3ζ transfected cells. Both mab reacted well with TREM-B1 (Fig 2A), but did not cross-react with the other TREM family members (Fig 2B and 2C). Surface expression of the respective proteins was validated by anti-FLAG staining.

### TREM-B1 is expressed on thrombocytes

**Fig 3:**
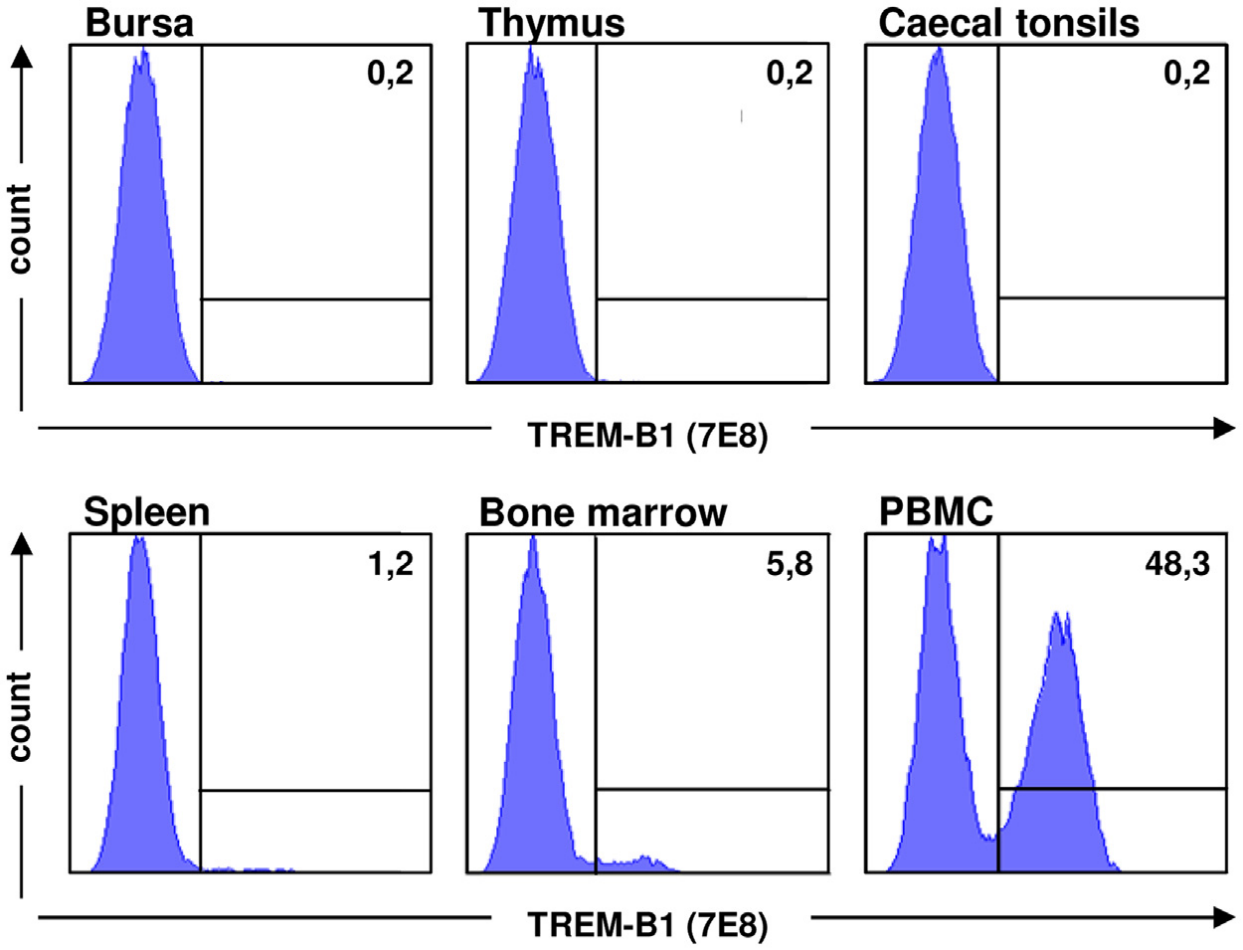
TREM-B1 is predominantly expressed on PBMC. Leukocytes obtained from bursa, thymus, caecal tonsils, spleen, bone marrow and blood (PBMC) were incubated with the 7E8 mab and analyzed by flow cytometry. The markers were set according to an isotype-matched negative control and the percentage of positive cells is indicated as a number. One of three representative experiments is shown.

**Fig 4:**
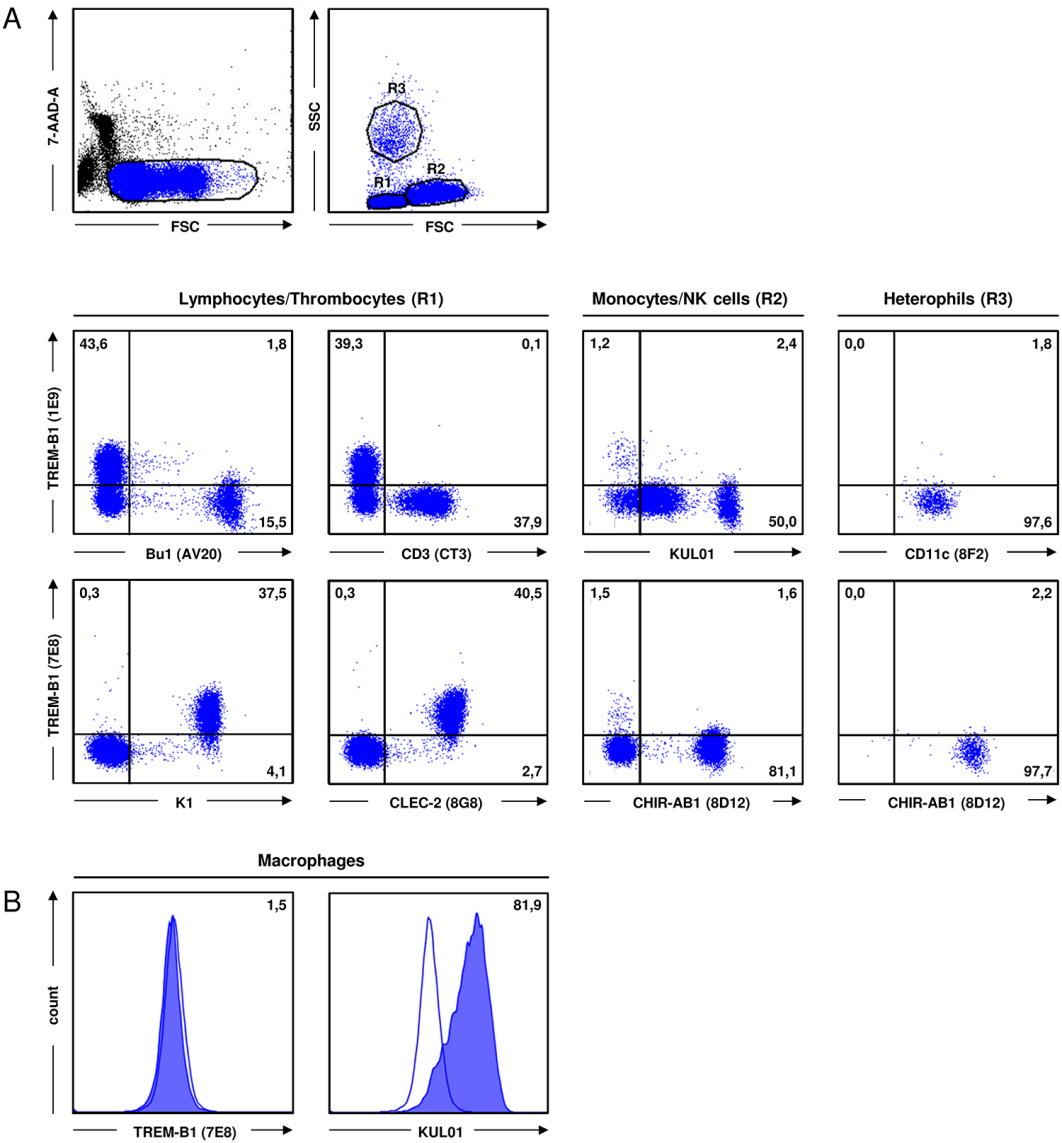
TREM-B1 is expressed on thrombocytes. (A) Viable PBMC were discriminated by 7-AAD staining and gated on lymphocytes/thrombocytes (R1), monocytes/NK cells (R2) or heterophils (R3) in FSC/SSC. Cells were double immunofluorescence stained with either the 1E9 (upper panel) or the 7E8 (lower panel) mab in combination with several cell surface markers as indicated. Numbers indicate the percentage of cells in the respective quadrants. Isotype-matched controls were included in each experiment. One of ten representative experiments is shown. (B) Macrophages were single stained with the TREM-B1 specific mab 7E9 and the macrophage/monocyte specific marker KUL01 (filled histograms). Isotype-matched controls are shown as open histograms and percentages of positive cells are indicated as numbers.

To investigate TREM-B1 protein expression, cell surface staining with the 7E8 mab on leukocyte preparations obtained from different tissues was conducted. Dead cells were stained with 7-AAD and excluded from the analysis. Lymphocytes from bursa, thymus and caecal tonsils showed no reactivity (Fig 3, upper panels). Only slight expression was found on cells from spleen and bone marrow and the highest expression was found on PBMC (Fig 3, lower panels), which is in accordance with real-time RT-PCR analysis (S1 Fig). Based on these results, double immunofluorescence staining on PBMC with antibodies specific for different cell populations was performed. Depending on the isotype of these antibodies, either the 1E9 or the 7E8 mab was applied. Living cells were identified by 7-AAD staining and either gated on lymphocytes/thrombocytes (R1), monocytes/NK cells (R2) or heterophils (R3) in FSC/SSC (Fig 4A). PBMC preparations of ten different animals were analyzed. Using the thrombocyte markers K1 or 8G8, all thrombocytes were found to be positive for TREM-B1 (Fig 4A, R1 gate, lower panels). The number of thrombocytes in the different preparations varied from 33.7% to 77.6%. In contrast, staining of lymphocytes (Fig 4A, R1 gate, upper panels), monocytes and NK cells (Fig 4A, R2 gate) and heterophils (Fig 4A, R3 gate) yielded no double positive population. Further analyses with the thrombocyte marker 23C6, the MHCII specific mab 2G11 and the 8C7 mab recognizing SLAMF4 (CD244) on thrombocytes, monocytes, NK cells and subsets of T cells and B cells, confirmed TREM-B1 expression is restricted to thrombocytes (data not shown).

To investigate the slight TREM-B1 expression on cells from spleen and bone marrow, these cells were also examined by double staining with the same antibody panel tested on PBMC (except 2G11 and 8C7). In compliance with PBMC staining, again thrombocytes were identified to be the only TREM-B1 expressing population (data not shown).

In addition, monocyte-derived macrophages were analyzed for TREM-B1 expression, but showed no reactivity (Fig 4B). In accordance with this, the chicken macrophage cell lines HD11 and BM-2 were also negative for TREM-B1 (data not shown).

**Fig 5:**
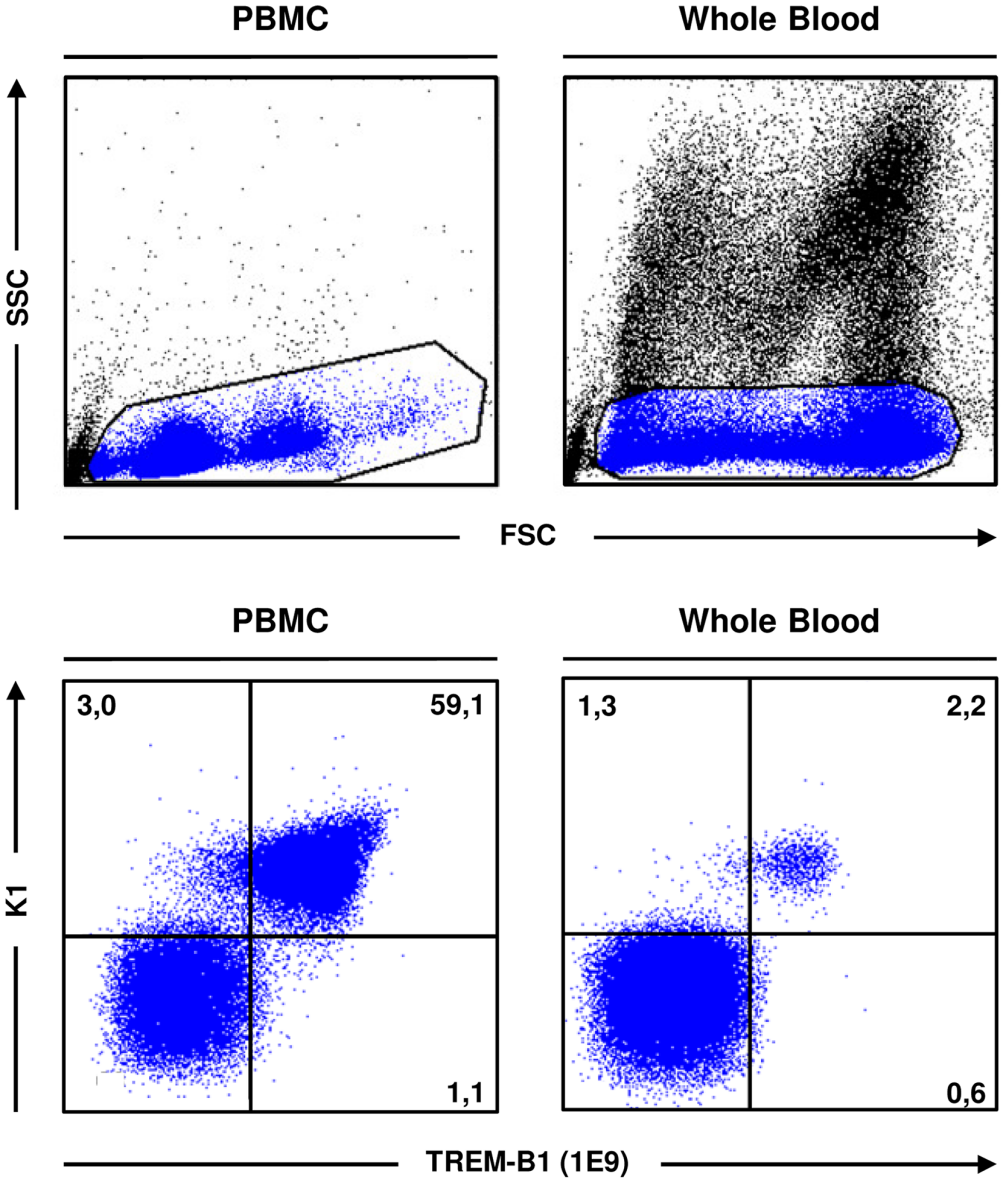
Cell preparation has no impact on the expression level of TREM-B1. PBMC obtained by density centrifugation and whole blood were double stained with the mab 1E9 and K1 (specific for monocytes and thrombocytes), gated on the entire leukocyte population by FSC/SSC and analyzed by flow cytometry. Numbers indicate the percentage of cells in the respective quadrants. The marker quadrants were set according to the unstained cells.

Thrombocytes can be easily activated. In order to check the impact of cell density centrifugation on thrombocyte protein expression level of TREM-B1, whole blood was analyzed using the Alexa Fluor 647-conjugated 1E9 mab combined with a phycoerythrin-conjugated K1 antibody and compared with thrombocytes prepared by density centrifugation. The entire leukocyte population of both PBMC and whole blood was gated by their light scatter characteristics in FSC/SSC (Fig 5, upper panels) and analyzed by flow cytometry. However, there was no difference in TREM-B1 expression level (Fig 5, lower panels).

### TREM-B1 is expressed as a glycosylated monomer

**Fig 6:**
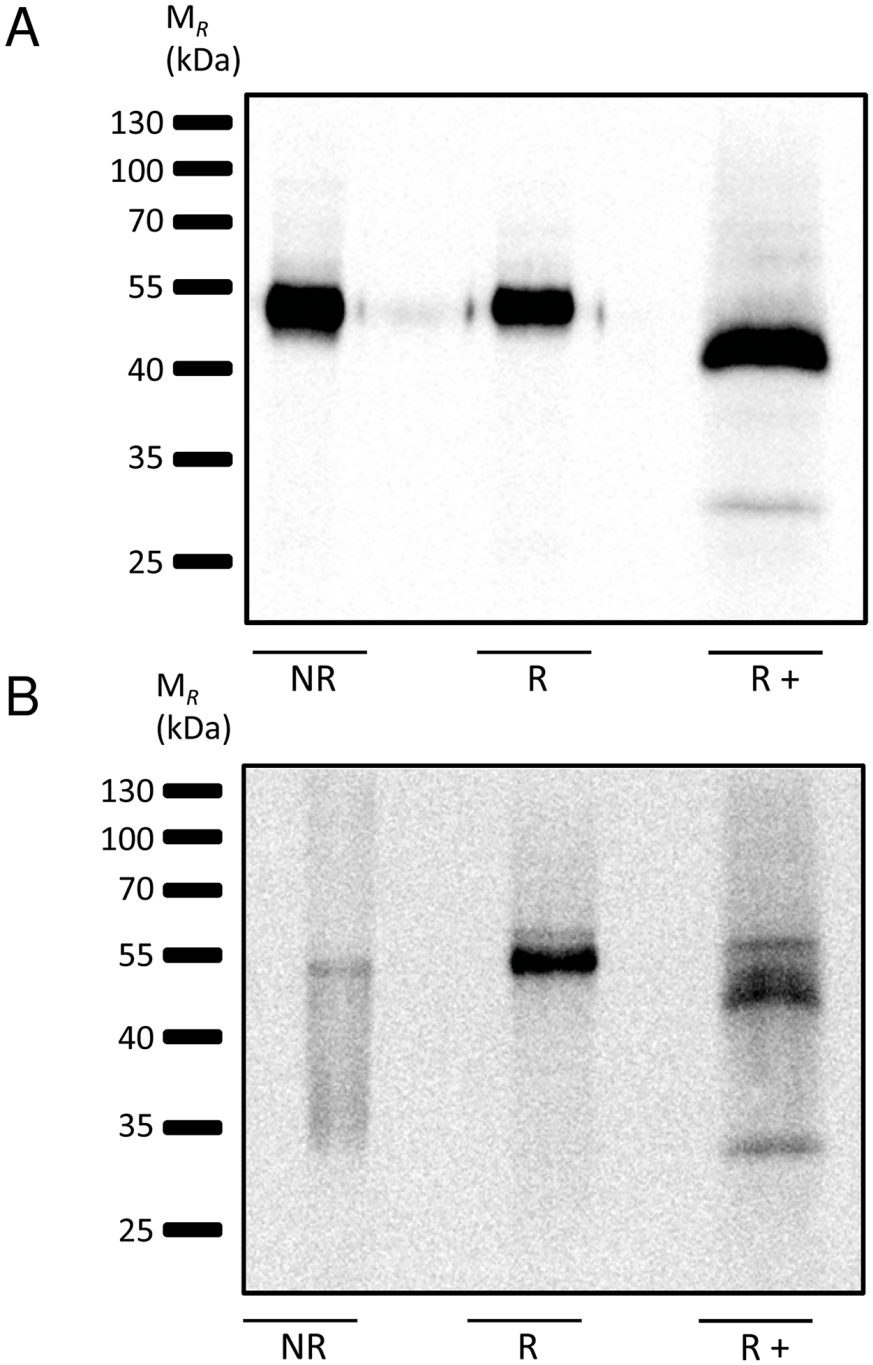
TREM-B1 is expressed as a glycosylated monomer. 2D8 cells stably transfected with a TREM-B1-FLAG construct (A) and PBMC (B) were surface biotinylated, lysed and immunoprecipitated with the 1E9 mab. Deglycosylation was performed with PNGaseF (+) and probes were analyzed under nonreducing (NR) and reducing (R) conditions. Following PAGE, size-fractioned proteins were blotted and detected with a streptavidin-HRP conjugate.

For biochemical analyses of TREM-B1, 2D8 cells expressing TREM-B1-FLAG were surface biotinylated, immunoprecipitated by the anti-TREM-B1 mab 1E9 and an irrelevant isotype control and subsequently analyzed by western blot. Under non-reducing conditions, a protein band of approximately 50 kDa was detected by a streptavidin-HRP conjugate (Fig 6A). Reduction of the protein yielded a single band on the same level and the addition of PNGase F for deglycosylation resulted in a molecule band with a reduced M_*R*_ of about 45 kDa, which is in accordance with the theoretical M_*R*_ of 46 kDa (Fig 6A). Therefore, TREM-B1 seems to be expressed as a glycosylated monomer. Immunoprecipitates of the isotype control showed no detectable protein (data not shown).

We performed similar experiments on primary PBMC and detected the same protein bands for native TREM-B1 (Fig 6B).

### TREM-B1 recruits SHP-2 upon phosphorylation

**Fig 7:**
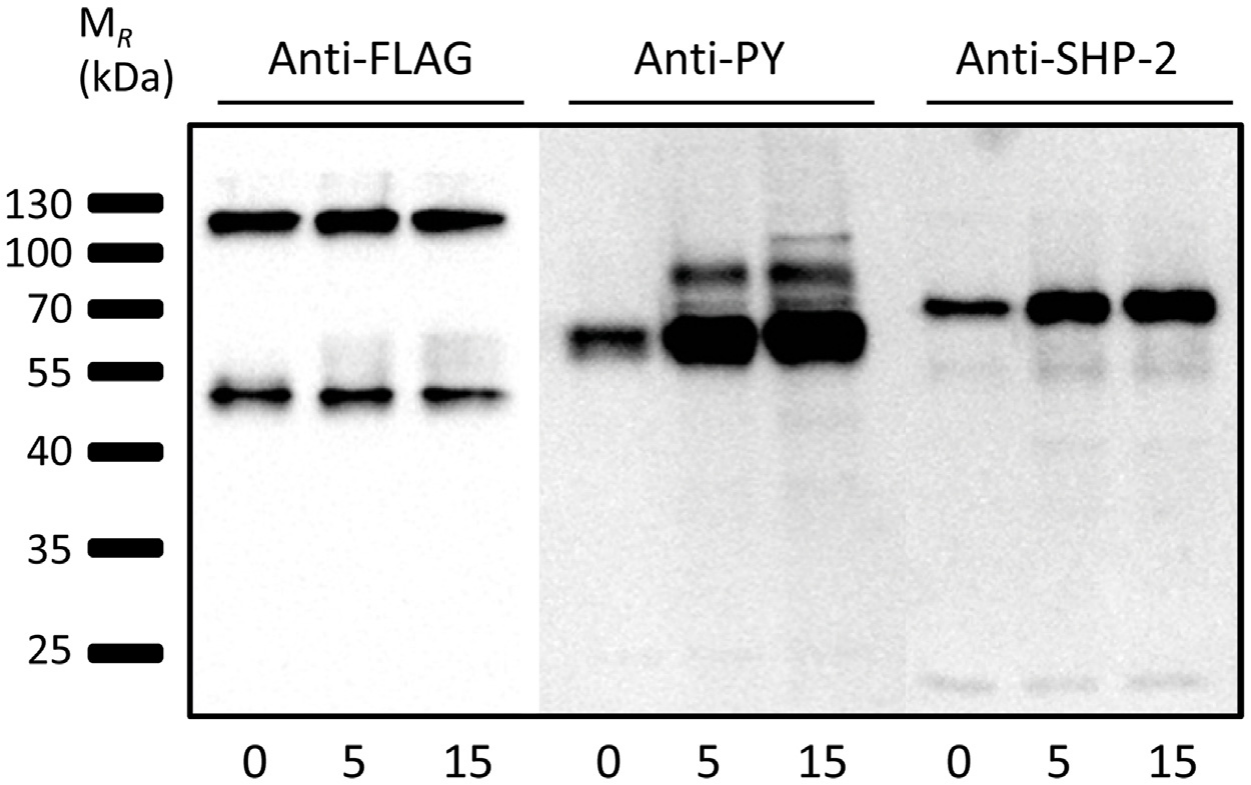
TREM-B1 recruits SHP-2 upon phosphorylation. TREM-B1-FLAG transfected 2D8 cells were treated with pervanadate for the times indicated, lysed and immunoprecipitated using an anti-FLAG mab. Indicated antibodies were used for detection of the proteins.

TREM-B1 contains three cytoplasmic signaling motifs, one ITSM and two ITIMs. Following phosphorylation, downstream signaling of the receptor might include protein phosphatases. Therefore, 2D8 cells expressing TREM-B1-FLAG were treated with pervanadate for 0, 5 and 15 minutes, respectively, and immunoprecipitated using anti-FLAG beads. The immunoblots were probed with a monoclonal antibody detecting phosphotyrosine and an antiserum directed against SHP-2. Cells treated for 0 minutes showed a weak basal level of phosphorylated tyrosine residues as well as weak association with SHP-2 which increased after pervanadate treatment (Fig 7). Equal protein loading was proven by using an anti-FLAG-HRP conjugate for detecting TREM-B1 with an apparent mass of 50 kDa (Fig 7).

### TREM-B1 crosslinking reduces CLEC-2 induced thrombocyte degranulation

**Fig 8:**
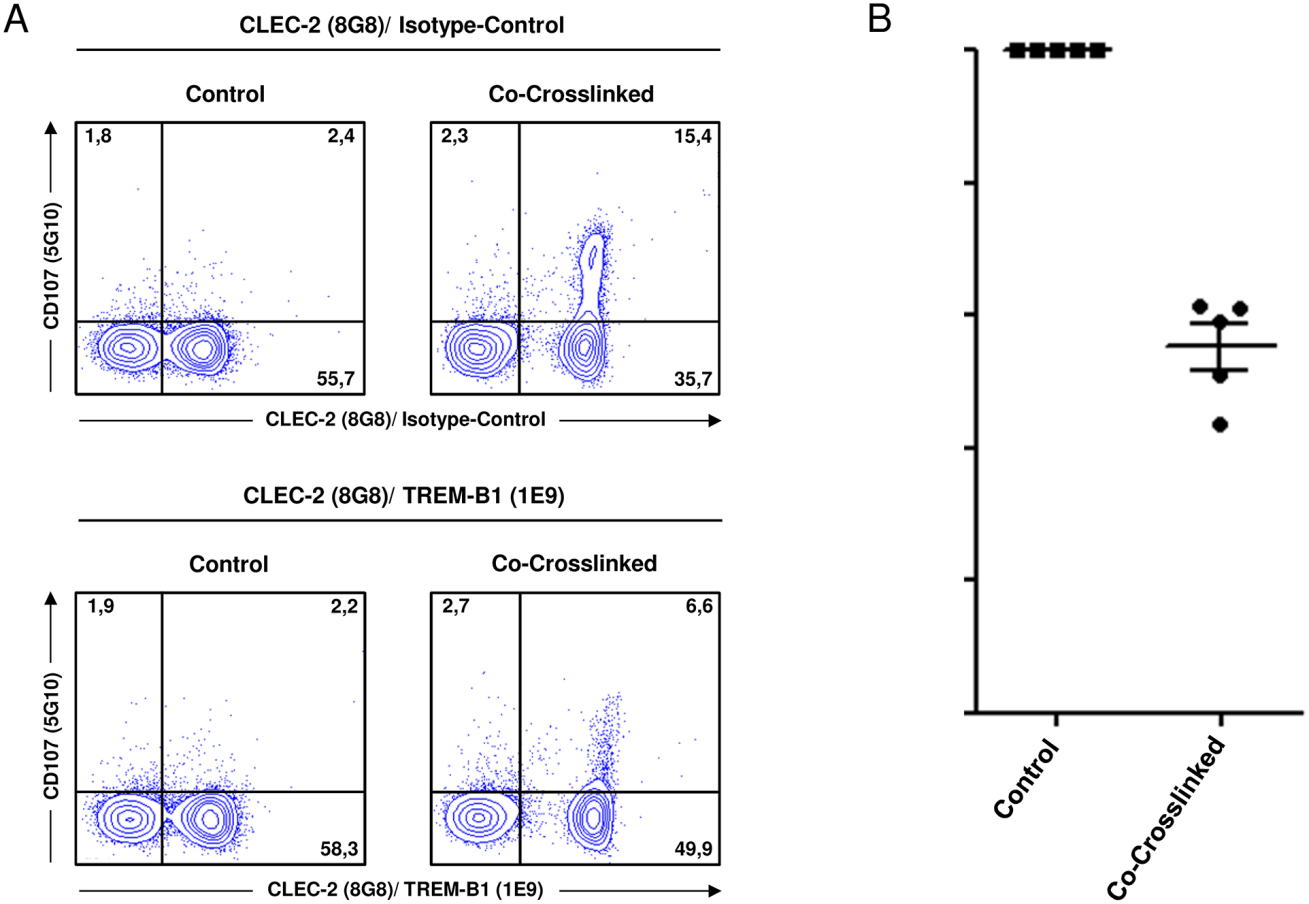
TREM-B1 crosslinking reduces CLEC-2 induced thrombocyte degranulation. (A) Co-crosslinking of CLEC-2 and TREM-B1 with the thrombocyte specific mab 8G8 and 1E9 decreased degranulation (lower panels) compared to co-crosslinking an irrelevant antibody and 8G8 (upper panels). Controls were incubated without crosslinking and subsequent stained with the respective antibodies. Numbers indicate the percentage of cells in the respective quadrants. One of five representative experiments is shown. (B) Percentage of TREM-B1 induced inhibition of CLEC-2 mediated thrombocyte degranulation. Markers represent single values of five different animals. Long bars represent the mean. Short bars represent the standard error of the mean (SEM).

Further examination of the inhibitory properties of TREM-B1 was performed by using the CD107 degranulation assay. Chicken thrombocytes can be activated by CLEC-2 crosslinking, resulting in degranulation with cell surface expression of the lysosomal protein CD107 [30]. PBMC were co-incubated with the thrombocyte specific mab 8G8 and 1E9 in the presence of a crosslinking secondary mab and degranulation was monitored by using an antibody specific for CD107. Dead cells were stained with 7-AAD and excluded from the analysis. 30 minutes after co-crosslinking, CD107 expression on the entire thrombocyte population was decreased compared to the isotype-matched control (Fig 8A, right panels). Controls without crosslinking did only show a slight CD107 expression (Fig 8A, left panels). In five animals, the reduction ranged from 43.7% to 61.3% (Fig 8B). For example, in Fig 8A degranulation was reduced by 61.1%.

## Discussion

Members of the TREM family have been the focus of intense research in the past years. Individual mammalian TREM receptors have been found to play a pivotal role in various functions and diseases including hemostasis and sepsis. The spectrum of different functions is not surprising, since the individual TREM receptors share only limited homology and each show a distinctive expression pattern [37, 38]. Apart from human and mouse TREM, this family of receptors has not been studied in great detail. Our analyses of chicken TREM aims to get a better understanding in the phylogeny of this receptor family and putatively conserved functions. It is therefore very important to characterize the expression pattern of individual chicken TREM receptors and to get insight into their functional potential. In this study, we generated TREM-B1 specific monoclonal antibodies to characterize cell surface expression patterns and biochemical properties. Combination of anti-TREM-B1 mab with a variety of different cell surface markers specific for chicken leukocyte subpopulations showed a TREM-B1 expression restricted to chicken thrombocytes. These thrombocytes were prepared by cell density gradient centrifugation from heparinized blood. To exclude unspecific activation of chicken thrombocytes using this separation method, which could potentially induce cell surface expression of TREM-B1, we also used whole blood preparation which implies a minimum of blood manipulation to exclude any accidental thrombocyte activation. Interestingly, we found no difference in expression levels comparing the two methods. Furthermore, we also stimulated purified thrombocytes with LPS to test potential up- or downregulation of TREM-B1 surface expression, but TREM-B1 expression levels were identical with or without stimulation (data not shown) indicating a constitutive expression of TREM-B1 on the cell surface of chicken thrombocytes.

The closest homologue to chicken TREM-B1 in mammals is the TREM-like transcript-1 (TLT-1), which is a member of the TREM multigene family on human chromosome 6p21 and mouse chromosome 17C [39–41]. It consists of a single V-set Ig-domain, an uncharged transmembrane region and a long cytoplasmic tail with one ITSM and one ITIM [42]. Whereas chicken thrombocytes display a constitutive TREM-B1 expression, mammalian platelets and megakaryocytes store TLT-1 in their α granules. TLT-1 surface expression can only be observed after platelet activation with thrombin, collagen or LPS [43, 44]. In 2009, fibrinogen was identified as the natural ligand for TLT-1 and TLT-1 facilitated platelet aggregation, indicating a function in blood hemostasis [45].

Moreover, soluble TLT-1 was detected in supernatants of activated murine and human platelets [46]. It was also identified in human serum and plasma and correlated to disseminated intravascular coagulation scores during sepsis [45]. Subsequent investigations showed that soluble TLT-1 is involved in the inhibition of leukocyte activation during sepsis. As a decoy receptor it competes against TREM-1 for binding to its ligand. This reduces TREM-1 induced pro-inflammatory cytokine production of neutrophils [47]. Soluble TLT-1 could be thus of therapeutic use against sepsis associated inflammation by down regulating the effect of TREM-1 in the immune response.

Two different sources of soluble TLT-1 are discussed in the literature. There is an alternatively spliced variant of TLT-1, which encodes only for the extracellular domain [45], but soluble TLT-1 is also shed from the surface of activated platelets [46].

Currently, we are investigating the presence of a soluble TREM-B1 in chicken. We have some evidence that there is an alternatively spliced soluble variant. Moreover, we will also examine the presence of soluble TREM-B1 shed from the surface of thrombocytes.

In addition to the cell surface expression, the specific anti-TREM-B1 mab 1E9 was used for immunoprecipitation of TREM-B1 in both stable transfected TREM-B1-FLAG 2D8 cells and chicken PBMC containing many thrombocytes. Subsequent western blot analysis under non-reducing and reducing conditions and after deglycosylation showed that TREM-B1 is expressed as a glycosylated monomer. TREM-B1-FLAG 2D8 cells were further used to analyze the signaling capabilities of the cytoplasmic region of TREM-B1. There are three tyrosine residues, which are embedded in either an ITSM (TIYAAI) or two ITIMs (VMYVNI and VEYATL) [20]. Pervanadate treatment and subsequent western blot analysis showed that these tyrosine residues are phosphorylated and the tyrosine specific phosphatase SHP-2 is recruited (Fig 7). Similarly to these results, human TLT-1 has also one ITSM (TTYTSL) and on ITIM (VTYATV) [39], as does have murine TLT-1 (ITSM: SIYTGS, ITIM: VTYATV) [42]. Initial experiments showed recruitment of the tyrosine specific phosphatase SHP-1 indicating an inhibitory potential of the receptor [43]. Another group showed recruitment of SHP-2, which did not result in inhibition, but in an enhancement of Fc receptor mediated Ca++ release in RBL cells [48] indicating coactivating potential of TLT-1. Furthermore, Washington et al showed an interaction of the cytoplasmic region of TLT-1 with cytoskeleton associated ERM proteins which would contribute to platelet aggregation [45].

Chicken thrombocytes and mammalian platelets differ in various aspects, in particular, since thrombocytes are nucleated cells. Little is known regarding their immune function but they react to LPS and express Toll like receptors [49–51]. Additionally, we and other groups demonstrated the expression of immune relevant cell surface receptor on chicken thrombocytes like CLEC-2, SLAMF4, TREM-A1, CD40L, CD200R-S1 and ggFCR [21, 22, 30, 52–54]. All these findings indicate that chicken thrombocytes might be involved in various types of immune responses.

We used the ability of CLEC-2 inducing degranulation of thrombocytes [30] for functional analysis of the potentially inhibitory TREM-B1. By this method we showed that co-crosslinking of TREM-B1 with CLEC-2 decreased thrombocyte degranulation by about 40–60%. This finding shows that TREM-B1 can act as a true inhibitory receptor.

As a next step to further reveal the TREM-B1 function on thrombocytes, we will focus on the identification of its natural ligand. For this purpose the TREM-B1 expressing reporter cell line BWZ.36 will be employed. This reporter assay has already been successfully used by our group in the identification of various ligands for different chicken Ig-like receptors [13, 23, 52, 55, 56].

In conclusion, analysis of the chicken TREM-B1 protein expression revealed expression on thrombocytes, which is similar to its mammalian homologue TLT-1. But chicken TREM-B1 is constitutively expressed on thrombocytes, while mammalian TLT-1 is only relocated to the cell surface upon platelet activation. Furthermore, TREM-B1 has been shown to inhibit thrombocyte degranulation. This may indicate a different physiological role of TREM-B1 on chicken thrombocytes.

## Supporting Information

S1 Fig TREM-B1 is highly expressed by PBMC. RNA from indicated tissues was analyzed for TREM-B1 expression by real-time RT-PCR using oligonucleotides summarized in Table 2. Cycle threshold values were normalized on 18S RNA and calibrated on liver using 2^-ΔΔCt^ formula. One representative out of three experiments is shown.
(TIF)
