# Supplementary figures and images for: Chicken TREM-B1, an Inhibitory Ig-Like Receptor Expressed on Chicken Thrombocytes
Source: PLoS One. 2016 Mar 11;11(3):e0151513. doi: 10.1371/journal.pone.0151513 (PMC4788293; doi:10.1371/journal.pone.0151513)

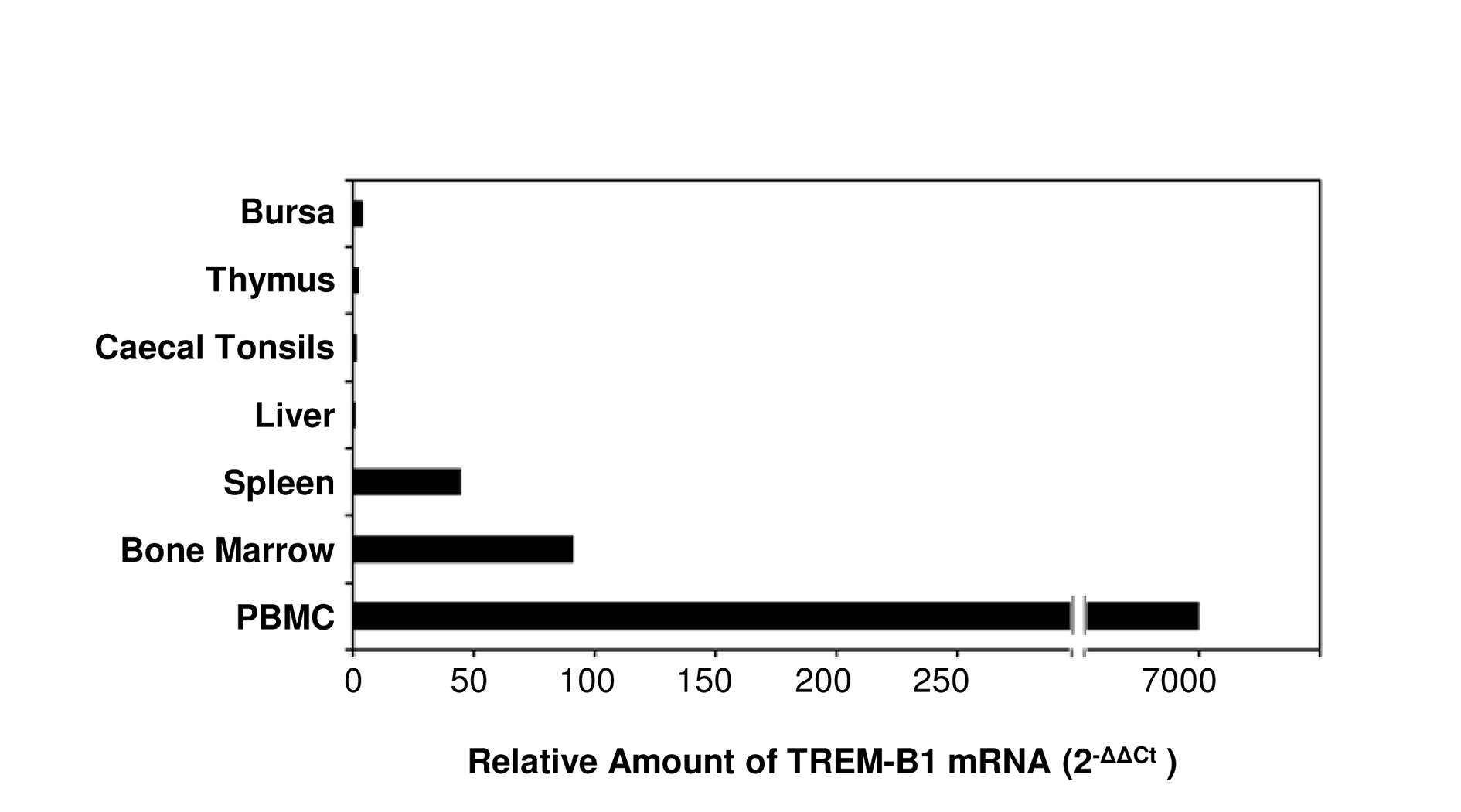

Supplement: S1 Fig — RNA from indicated tissues was analyzed for TREM-B1 expression by real-time RT-PCR using oligonucleotides summarized in Table 2. Cycle threshold values were normalized on 18S RNA and calibrated on liver using 2-ΔΔCt formula. One representative out of three experiments is shown. (TIF) [file pone.0151513.s001.tif]
